# Sources of Variation in the Gut Microbial Community of *Lycaeides melissa* Caterpillars

**DOI:** 10.1038/s41598-017-11781-1

**Published:** 2017-09-12

**Authors:** Samridhi Chaturvedi, Alexandre Rego, Lauren K. Lucas, Zachariah Gompert

**Affiliations:** 1 Utah State University, Department of Biology, Logan, 84322 UT USA; 2 Utah State University, Ecology Center, Logan, 84322 UT USA

## Abstract

Microbes can mediate insect-plant interactions and have been implicated in major evolutionary transitions to herbivory. Whether microbes also play a role in more modest host shifts or expansions in herbivorous insects is less clear. Here we evaluate the potential for gut microbial communities to constrain or facilitate host plant use in the Melissa blue butterfly (*Lycaeides melissa*). We conducted a larval rearing experiment where caterpillars from two populations were fed plant tissue from two hosts. We used 16S rRNA sequencing to quantify the relative effects of sample type (frass versus whole caterpillar), diet (plant species), butterfly population and development (caterpillar age) on the composition and diversity of the caterpillar gut microbial communities, and secondly, to test for a relationship between microbial community and larval performance. Gut microbial communities varied over time (that is, with caterpillar age) and differed between frass and whole caterpillar samples. Diet (host plant) and butterfly population had much more limited effects on microbial communities. We found no evidence that gut microbe community composition was associated with caterpillar weight, and thus, our results provide no support for the hypothesis that variation in microbial community affects performance in *L*. *melissa*.

## Introduction

Despite the low nutrient content, indigestibility and toxicity of many plant tissues, plant-feeding insects are among the most abundant and diverse groups of organisms on Earth^1^. Herbivorous insects possess numerous morphological, behavioral and physiological traits that allow them to overcome these dietary obstacles^2^. Insect species and populations are often highly specialized^3, 4^, feeding on one or a few families or even species of plants. Therefore, evolutionary shifts to new plant hosts can lead to speciation and catalyze further diversification^5–9^. Specific adaptations that allow insects to utilize novel plant hosts have been identified, and include changes in the structure or abundance of gut enzymes that reduce the toxicity of plant allelochemicals^10–12^. Changes in gut microbial communities could facilitate host plant shifts in a similar manner^13^, but data in support of this are mostly lacking^14–17^.

Vertically transmitted microorganisms provide necessary nutritional benefits for some insects that have poor or unbalanced diets, such as phloem-sap or wood feeders^18–20^. For example, microorganisms could provision insects with essential amino acids which were missing in their diet^20^. Moreover, the acquisition of symbiotic microbes has been associated with major evolutionary shifts from non-plant-based to plant-based diets^16, 21^. Whether microbial symbionts facilitate more modest host-plant shifts, that is host shifts or expansions to novel plant species or genera, is less clear^16, 17^. Gut microbes in particular have been hypothesized to shape host use and diet breadth by allowing herbivorous insects to detoxify specific plant allelochemicals (hereafter the “gut microbial facilitation hypothesis”^14, 17^). The high diversity of catabolic pathways available to microbes and the potential for gut microbes to interact with plant toxins make the gut microbial facilitation hypothesis an intriguing possibility^17^. Perhaps the strongest support for this hypothesis comes from studies of wild populations of pea aphids that found an association between the presence of specific gut microbes and host use^22^. However, experimental tests of the effects of these microbes on pea aphid performance have been inconsistent^22–24^. More generally, the lack of empirical support for the gut microbe facilitation hypothesis could be the result of a paucity of experimental studies designed to test it^16, 17^, and additional evidence in support of this hypothesis is beginning to emerge^25^. Here we (i) quantify the contribution of different factors (i.e., host, population, development and sample type) to variation in the gut microbiome, and (ii) evaluate the gut microbial facilitation hypothesis in the Melissa blue butterfly (*Lycaeides melissa*).

*Lycaeides melissa* (Lepidoptera: Lycaenidae) occurs in the western US and southern Canada where it feeds exclusively on the leaves and flowers of legumes (Fabaceae); common native hosts include members of the *Astragalus* and *Lupinus* genera^26, 27^. Alfalfa (*Medicago sativa*) was introduced to the western US in the mid 1800s as a forage crop^28^, and has since been colonized by *L*. *melissa*. Despite evidence of adaptation to this novel resource, *M*. *sativa* remains a poor host relative to known native hosts^29–31^. For example, *L. melissa* butterflies reared on *M*. *sativa* are smaller and suffer higher larval mortality than caterpillars reared on native hosts^29, 31^. Indeed, population persistence on alfalfa in the wild may depend on the presence of mutualistic ants that tend *L*. *melissa* caterpillars^29^.

Variation exists within and among *L*. *melissa* populations for host acceptance and larval performance^29, 31^. Likewise, host-plant populations and species differ in their average palatability to *L*. *melissa* ^29, 32^. However, this variability in host acceptance and performance is poorly predicted by plant phytochemistry or protein content^32^. Microbial symbionts could explain some of this variation, and this is a focus of our current study. We consider only bacterial microbes at present, though complementary work investigating the role of fungal endophytes is underway^33^.

Microbes could influence *L*. *melissa* host plant use in several ways. If endophytic or epiphytic microbial communities vary among host plants and caterpillars acquire their gut microbiome from their diet (e.g., refs 21 and 34), butterflies feeding on different populations or species of plant should have different gut microbes. Additionally, genetic differences among individual butterflies or populations could affect the gut environment in such a way that favors different gut microbes and thereby alters the gut microbial community. In either case, the resulting differences in gut microbiomes could be beneficial, detrimental, or have no effect on *L*. *melissa* fitness and population persistence. If caterpillar gut microbiome is mostly determined by diet (i.e., if it has a low heritability), then the ecological consequences of microbes on a host shift would be immediate. Caterpillars would acquire a new microbial community upon colonizing a new population or species of plant and experience any fitness consequences that follow. Alternatively, if gut variation in microbiome has a substantial genetic component (i.e., a non-negligible heritability), shifts in gut microbiome could occur over multiple generations due to evolution by genetic drift or selection and could be a key component of host-associated adaptation and specialization.

Herein we describe a larval rearing experiment conducted to measure the effects of diet (host plant), population (a surrogate for genotype) and development on *L*. *melissa* caterpillar microbiomes. The experiment involved two *L*. *melissa* butterfly populations from northern Utah (USA): one in Blacksmith Fork Canyon, near Hardware Ranch (HWR; latitude = 41.6188°N, longitude = 111.5647°W) and one along the Bonneville Shoreline Trail (BST; latitude = 41.7428°N, longitude = 111.7885°W). We lack data on the genetic similarity of these butterfly populations, but results from large genomic surveys of many *Lycaeides* populations show that even populations separated by short distances are genetically differentiated^35^. More importantly, *L*. *melissa* feeds on alfalfa (*Medicago sativa*) and a native lupine (*Lupinus argenteus*) at HWR, whereas alfalfa is the only available host at BST (personal obs.). Thus, with respect to host plant, these sites represent different selective environments that could drive adaptive genetic-based differences in gut microbiomes. At HWR, the two host plant species are intermixed and butterflies can readily fly from one plant species to the other while laying eggs. Dispersal in caterpillars is much more limited.

Microbial communities were measured from plants, caterpillars and frass (caterpillar excrement) using high-throughput DNA sequencing of 16S rRNA. Because *L*. *melissa* at these two populations differ in host use, host-associated selection could differ between sites and lead to local adaptation, which could include adaptive differences in the caterpillar gut environment and consequently in gut microbial communities, or gut microbial communities could be determined mostly by diet and not be affected by genetic differences between these populations. We tested these alternatives. We also tested for effects of microbial community composition and diversity on caterpillar performance (i.e., caterpillar weight). We show that caterpillars harbor a microbial community that varies over time, and that is minimally affected by source population or diet. We fail to find compelling evidence for an association between microbial community composition and larval performance in general, or in a host-specific manner.

## Results

### Microbial community structure

**Figure 1:**
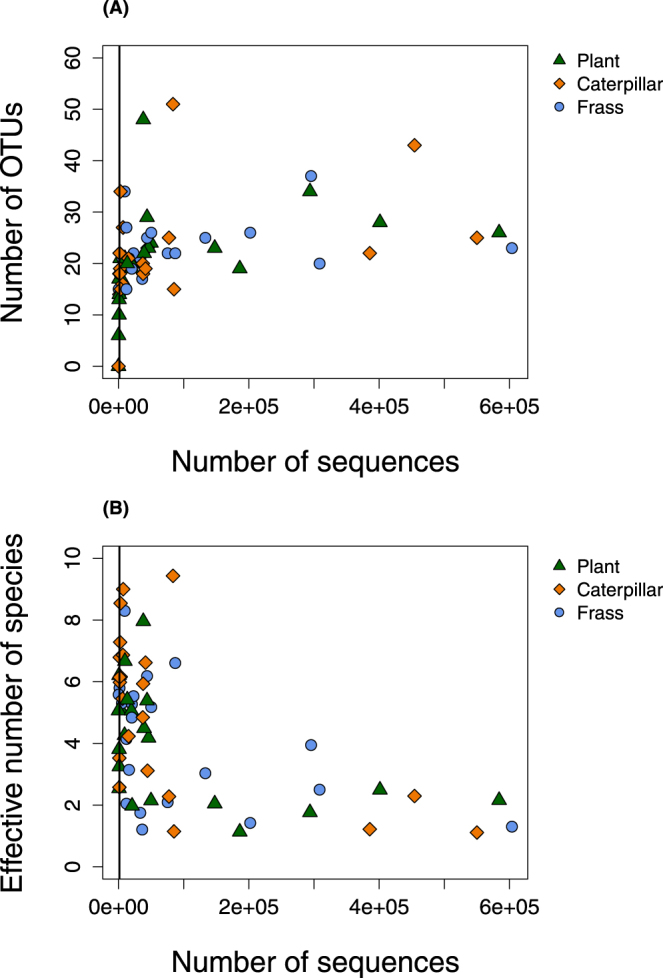
OTU richness and diversity. Scatterplots show the (**A**) number of OTUs and (**B**) effective number of species (^2^ *D*) for each sample as a function of sequencing depth prior to rarefaction but after removing chloroplast, mitochondrial and *Wolbachia* sequences. Colors and symbols denote different sample types and vertical lines show the rarefaction cutoff for each sample for downstream analysis (1311 sequences).

**Figure 2:**
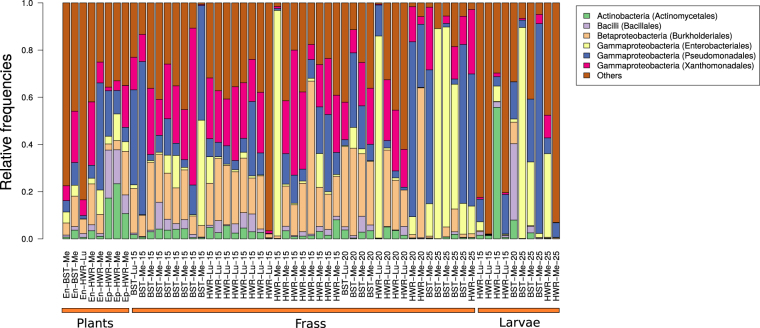
Relative bacterial abundances. Relative abundances of the major microbial taxa in the plant, larval (caterpillar) and frass samples, calculated from operational taxonomic unit (OTU) counts. Samples are sorted according to sample type (plant, whole caterpillar or frass), population, plant and larval age. Sample abbreviation are: En = endophytes; Ep = epiphytes; Hardware Ranch = HWR, Bonneville Shoreline Trail = BST; Me = (*M*. *sativa*), and Lu = (*L*. *argenteus*). Numbers (15, 20 or 25) indicate caterpillar age. In the legend, OTU are identified as class (order).

**Figure 3:**
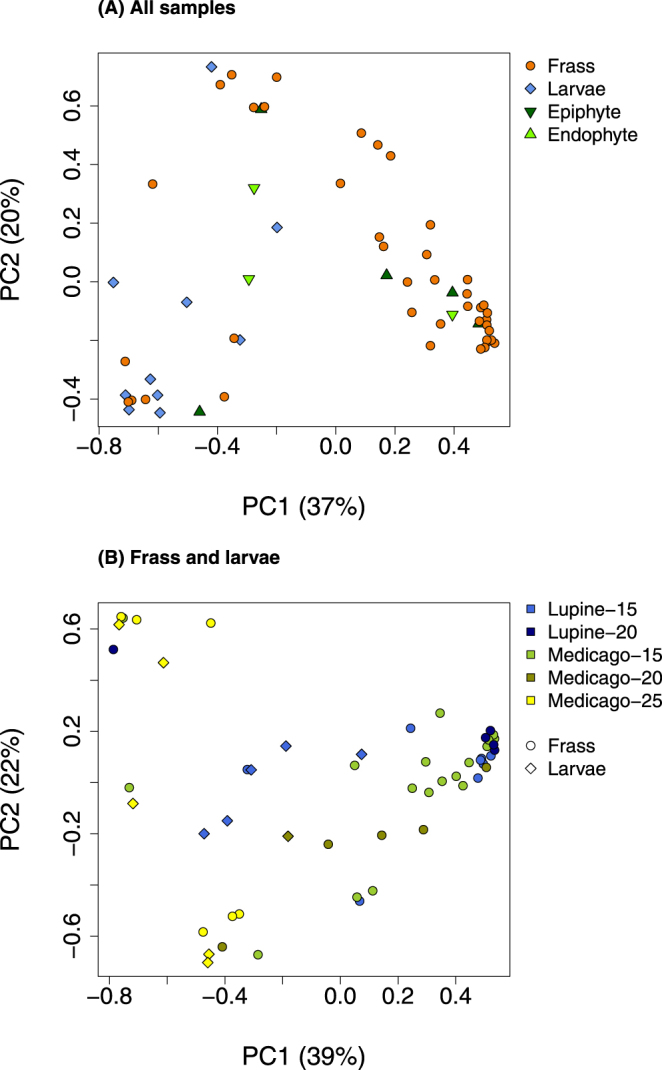
Principal component analysis. Scatterplots show an ordination of microbial communities from chord-transformed relative abundance data for (**A**) all samples, and (**B**) frass and larvae. Colors and symbols denote different treatments and sample types (see legends).

**Figure 4:**
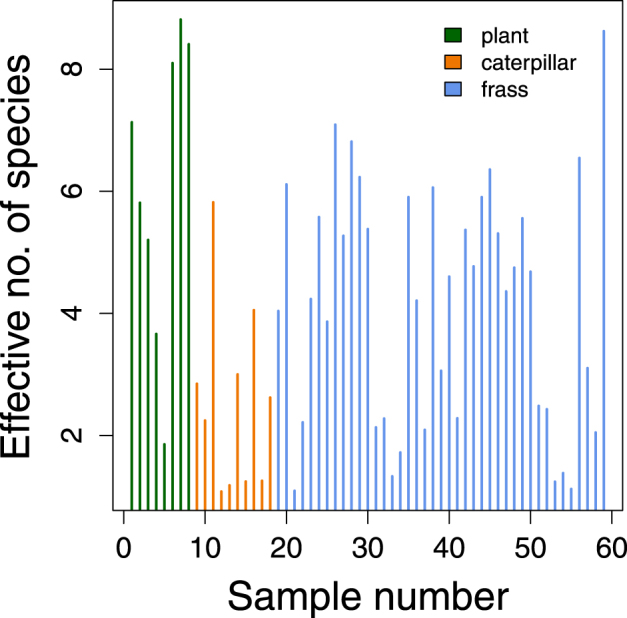
Phylotype diversity. True phylotype diversity, that is Hill’s effective species number with *q* = 2, is shown for all plant, caterpillar and frass samples.

After removing chloroplast and mitochondrial sequences and performing rarefaction, we retained 59 samples (frass = 41, caterpillar = 10 and plant [endophytes and epiphytes] = 8) (sequence depth prior to rarefaction was not strongly associated with measures of OTU richness or diversity in the samples; Fig. 1). Microbial communities from frass, whole caterpillar and plant samples were mostly dominated by Betaproteobacteria, Gammaproteobacteria, Actinobacteria and Firmicutes (order Bacilli) (Fig. 2). The first two principal components (PCs) of the chord-transformed relative abundance matrix captured most of the variation in microbial community composition among the caterpillar, frass and plant samples (57% of the total variation) (Table S1; Fig. 3a). Caterpillar, frass and plant microbial communities overlapped in PC space, but sample types differed in their average PC scores and degree of variability (Table 1). Most notably, average caterpillar communities differed from frass and plant communities with respect to PC1 scores (Bayesian posterior prob. [pp] *μ*_Larvae_ > *μ*_Frass_ > 0.99; pp *μ*_Larvae_ > *μ*_Plant_ > 0.99). Frass and plant microbe communities were generally more similar. Complementary analyses based on a principal coordinate analysis (PCOA) of Bray-Curtis community dissimilarities gave similar results (PCs and PCOs were highly correlated: |*r*_*PC*1,*PCO*1_| = 0.98, |*r*_*PC*2,*PCO*2_| = 0.64, both *p* < 0.0001; Table 1; Fig. S1a). The effective number of phylotypes (that is “true diversity” = ^2^ *D*) was higher and differed more among samples for the plant microbial communities (mean ^2^ *D*, posterior median [pm] = 6.13, 95% ETPIs = $[4.09,\,8.06]$; s.d. ^2^ *D*, pm = 2.59, 95% ETPIs $[1.63,\,4.97]$) than the caterpillar microbial communities (mean ^2^ *D*, pm = 2.54, 95% ETPIs = $[1.48,\,3.64]$; s.d. ^2^ *D*, pm = 1.58, 95% ETPIs = $[1.05,\,2.75]$) (Fig. 4). Intermediate diversity levels were observed in the frass microbial communities (mean ^2^ *D*, pm = 4.14, 95% ETPIs = $[3.52,\,4.77]$; s.d. ^2^ *D*, pm = 1.98, 95% ETPIs = $[1.62,\,2.51]$).

**Table 1:** Bayesian estimates of microbial community composition for different sample types.

Parameter	PCO1		PCO2		PC1		PC2	
	pm	ETPIs	pm	ETPIs	pm	ETPIs	pm	ETPIs
*μ*_Plant_	0.057	−0.092, 0.209	−0.056	−0.197, 0.091	0.062	−0.239, 0.358	0.013	−0.213, 0.238
*μ*_Frass_	0.074	−0.011, 0.162	0.063	0.018, 0.107	0.121	−0.011, 0.250	0.032	−0.077, 0.135
*μ*_Larvae_	−0.356	−0.471, −0.245	−0.205	−0.407, 0.000	−0.549	−0.679, −0.414	−0.139	−0.408, 0.137
*σ*_Plant_	0.205	0.133, 0.375	0.202	0.130, 0.373	0.401	0.260, 0.724	0.310	0.200, 0.568
*σ*_Frass_	0.278	0.225, 0.351	0.143	0.116, 0.181	0.423	0.344, 0.540	0.331	0.268, 0.420
*σ*_Larvae_	0.163	0.108, 0.291	0.299	0.198, 0.522	0.194	0.128, 0.337	0.389	0.258, 0.689

Posterior medians (‘pm’) and 95% ETPIs are provided for the mean (*μ*) and standard deviation (*σ*) of principal coordinate (PCO) and principal component (PC) scores.

Random Forest (RF) (a decision tree classification method) was able to correctly classify most frass and caterpillar samples (as frass and caterpillars, respectively), whereas most plant samples were incorrectly classified as frass (Table S2). Discrimination between frass and caterpillar samples was mostly due to the fact that *Wolbachia* (Order Rickettsiales) was very common in the whole caterpillar samples but largely absent from the frass samples (GINI index = 4.89; whole caterpillar relative abundance: mean = 0.37, s.d. = 0.36; frass relative abundance: mean = 0.0007, s.d. = 0.003; Table S3). This pattern is unsurprising, as *Wolbachia* is a common intracellular symbiont in arthropods and has been found in *Lycaeides melissa* ^36^ and other Lycaenid butterflies^37^.

### Determinants of frass and caterpillar microbial communities

**Figure 5:**
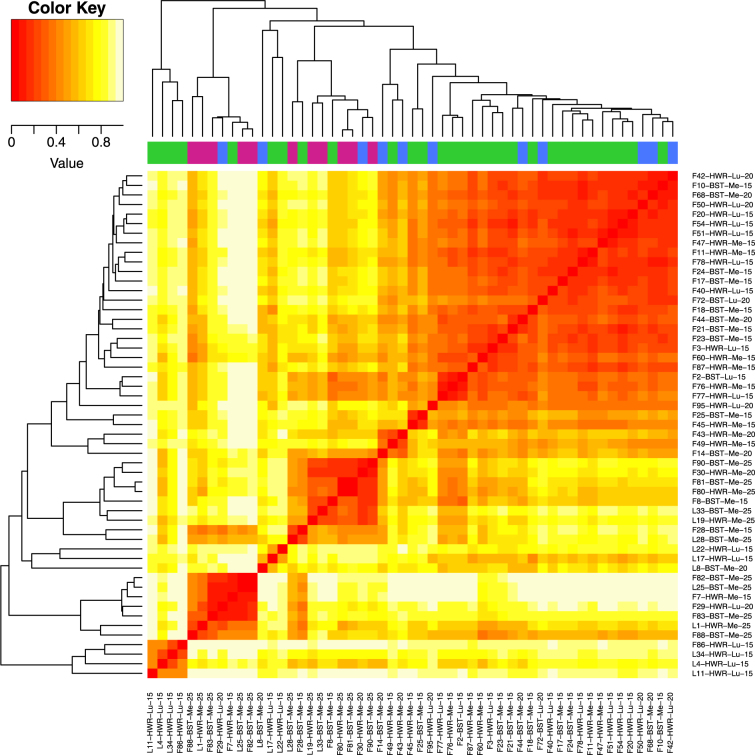
Hierarchical cluster analysis. Heatmap of Bray-Curtis dissimilarities between samples and corresponding dendrograms based on bacterial OTU abundances. Each row and column represents a sample. Cell colors indicate dissimilarity values between row and column microbial communities (red = greater similarity and yellow = less similarity). The dendrogram groups samples by hierarchical clustering based on microbial community similarity. Sample abbreviations: F = frass, L = larvae, HWR = Hardware Ranch, BST = Bonneville Shoreline Trail, Me = *M*. *sativa*, and Lu = *L*. *argenteus*. Numbers with F and L indicate sample IDs, whereas the final number in each ID gives the caterpillar age (15, 20, or 25). Color bars above the heatmap indicate the age of samples, (green = 15 days, blue = 20 days, purple = 25 days). The heatmap and dendrogram show that microbiomes from different sample types, different age caterpillars and different treatments do not form distinct groups or sub-cluster, but that there is a tendency for sets of similar samples (i.e., samples from the same age caterpillar) to be more similar and cluster together.

After removing the *Wolbachia* sequences and re-rarifying the OTU table, we retained 53 samples (42 frass and 11 whole caterpillars) for subsequent analyses of frass and caterpillar microbial communities. Hierarchical clustering of the frass and whole caterpillar samples based on differences in microbial communities did not show distinct clusters based on age, source population, host plant (diet) or sample type. However, several small clusters or groups in the dendrogram consisted of frass or whole caterpillars of the same age or reared on the same host-plant (Figs 5 and S2).

The first two PCs from an ordination of only the frass and whole caterpillar microbiomes (chord-transformed relative abundances) captured most of the variation in microbial community composition among these samples (61% of the total variation in the chord transformed relative abundances) (Fig. 3b). PC1 and PC2 reflected variation in the relative abundance of several Proteobacteria (Table S4). Bayesian linear models showed that microbial communities (as measured by PCs) were mostly affected by caterpillar age (days since hatching) and sample type (frass vs. whole caterpillar) (PCOs and PCs were highly correlated, |*r*_*PC*1,*PCO*1_| = 0.99, |*r*_*PC*2,*PCO*2_| = 0.98, both *p* < 0.0001, and thus analyses based on PCOs gave similar results; Fig. S1b). Specifically, even after removing *Wolbachia* sequences, frass and whole caterpillars contained different microbiomes (*β*_type_ for PC1, pm = −0.418, 95% ETPIs = $[-\,0.685,\,-0.150]$). The microbial communities of caterpillar guts (measured from frass and whole caterpillar samples) also changed over time with respect to PC1 scores (*β*_age_ for PC1, pm = −0.048, 95% ETPIs = $[\,-\,0.076,\,-0.020]$). Similarly, phylotype diversity was lower in frass and whole caterpillar microbial communities from older larvae (*β*_age_, pm = −0.151, 95% ETPIs = $[-\,0.296,\,-0.005]$). Based on these estimates, diversity dropped by almost two effective species between the 15 and 25 day samples, with the most pronounced shift occurring between the 20 and 25 days, that is, late in larval development (pupation occurred between 23 and 28 days of development). We failed to detect credible effects of butterfly population or plant species on community composition or diversity, and more generally, RF failed to accurately discriminate between samples from different sources (frass vs. whole caterpillar), host plant treatments, populations or samples collected at different larval ages (Tables S5, S6 and S7). Instead, RF generally assigned most samples to the more common group.

**Figure 6:**
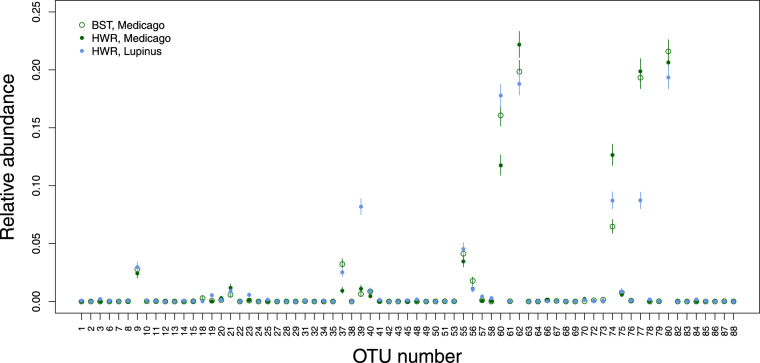
Microbe relative abundance from frass samples. Points and vertical bars denote Bayesian point estimates (posterior medians) and 95% ETPIs for the relative abundance of different microbial OTUs in 15 and 20 day frass samples. Colors and symbols denote samples from different plant (*L*. *argenteus* or *M*. *sativa*) and population (BST or HWR) treatments. Estimates are from a Bayesian multinomial-Dirichlet model. Low sample sizes precluded meaningful estimates for BST on *L*. *argenteus*. OTU numbers are defined in Table S9.

Despite the lack of a clear effect of butterfly population or host plant on community composition, we identified several microorganisms with significantly different relative abundances in different butterfly population × plant species treatment combinations. This was done using a Bayesian multinomial-Dirichlet for relative abundance counts and considering only frass samples from 15 or 20 day old larvae (we focused on this subset of samples to maximize the sample size while minimizing the confounding effects of caterpillar age and sample type documented above; Fig. 6). Lactobacillales (Bacilli) and Rhodospirillales (Alphaproteobacteria) were more abundant in frass samples from caterpillars reared on *L*. *argenteus* (Lactobacillales: pm, *L*. *argenteus*-HWR [L-HWR] = 0.082, *M*. *sativa*-HWR [M-HWR] = 0.011, *M*. *sativa*-BST [M-BST] = 0.006; Rhodospirillales: pms, L-HWR = 0.0030, M-HWR = 2.3*e* ^−4^, M-BST = 3.6*e* ^−5^), whereas Pseudomonadales (Gammaproteobacteria) were more abundant in frass from caterpillars fed *M*. *sativa* (pm, L-HWR = 0.087, M-HWR = 0.193, M-BST = 0.193). Rhizobiales (Alphaproteobacteria) were more abundant in frass from BST caterpillars (pm, L-HWR = 0.010, M-HWR = 0.011, M-BST = 0.18), and Enterobacteriales (Gammaproteobacteria) and Sphingomondadales (Alphaproteobacteria) differed in relative abundance based on host plant and butterfly population (Enterobacteriales: pm, L-HWR = 0.087, M-HWR = 0.013, M-BST = 0.065); Sphingomondadales, pm, L-HWR = 0.178, M-HWR = 0.117, M-BST = 0.161)). In each of these cases, the posterior probability for sample differences was ≥0.99, and the posterior predictive root-mean square error (RMSE) was significantly lower for a model allowing for different microbe relative abundances for each treatment combination than a constrained null model (pp = 0.969).

### Microbial community and larval performance

Thirty-one percent of the 181 caterpillars survived to 15 days, that is, to when the first frass samples were collected and larval weight was measured. We found greater evidence for an effect of population on survival than plant (*β*_pop_, pm = −0.56, 95% ETPIs = $[-1.22,\,0.07]$; *β*_plant_, pm = −0.15, 95% ETPIs = $[-\,0.84,\,0.51]$), such that probabilities of survival were 0.38 (95% ETPIs = 0.27, 0.50) for HWR caterpillars on *M*. *sativa*, 0.35 (95% ETPIs = $[0.22,\,0.49]$) for HWR caterpillars on *L*. *argenteus*, 0.26 (95% ETPIs = $[0.17,0.37]$) for BST caterpillars on *M*. *sativa*, and 0.23 (95% ETPIs = 0.13, 0.37) for BST caterpillars on *L*. *argenteus*.

We next tested for an association between microbial communities and caterpillar weight (a metric of performance). We focused on PC1, PC2, PCO1 and PCO2 (measures of community composition) and phylotype diversity (^2^ *D*) for frass samples from 15 and 20 day old caterpillars (that is, from caterpillars that survived long enough for the first frass samples to be taken; *N* = 31 samples). The best model (lowest DIC) was the base model with plant, population and age (i.e., with no effect of microbial community), but several other models that included effects of microorganisms had only slightly worse DIC values (Table S8). Unsurprisingly, caterpillar weight increased with age (*β*_age_, pm = 0.225, 95% ETPIs = $[0.098,\,0.353]$) in the base model. We found that feeding on *M*. *sativa* reduced caterpillar weight relative to feeding on *L*. *argenteus* (*β*_plant_, pm = −0.961, 95% ETPIs = $[-1.643,\,-0.281]$, pp < 0 = 0.99).

## Discussion

Simple models of genetic trade-offs have mostly failed to explain host specialization in herbivorous insects^31, 38, 39^. However, experimental tests have rarely considered symbionts, such as gut microbes, which can mediate interactions between insects and their hosts^16, 40, 41^. As proposed by the gut microbial facilitation hypothesis, microbial communities have to affect fitness and have a non-zero heritability for adaptive shifts in gut microbes to contribute to host use evolution by insects^17^. Our current study represents an initial attempt to evaluate evidence for and against this hypothesis (also see refs 25 and 42). We failed to find a convincing association between microbial community and larval performance. Instead we found that microbes mostly varied over time and differed between frass and whole caterpillar samples, with frass samples harboring microbiomes that were more similar to the plant microbial communities. We found minimal overall effects of butterfly population or diet (host plant) on gut microbiomes, but did identify several microorganisms that differed in their relative abundances across treatments. Thus, in total, our results do not suggest that genetic differences among *L*. *melissa* populations contribute substantially to adaptive variation in microbial communities (at least not for the populations we studied). Nonetheless, gut microbes could contribute to host use evolution in *L*. *melissa*, if for example, microbial variation among plants affects whether initial colonization of a new host is possible. Additionally, as we only considered a single pair of butterfly populations, genetic variation for microbial communities could certainly exist at greater spatial scales (i.e., between more distant populations via genetic drift or selection) or even among individuals within some populations. Thus, our current results neither strongly support nor refute the gut microbial facilitation hypothesis in *L*. *melissa*.

We discuss our results and these issues in more detail below, but first, three potential limitations of our study should be noted. First, we used microbial communities from frass as a proxy for the gut microbial communities in caterpillars. This approach has been used in previous studies of caterpillar gut communities^37, 43^ and allows for non-destructive sampling of community composition and diversity over time or developmental stages. But, even though frass and caterpillar communities were more similar after removing *Wolbachia* sequences, they still differed. This means that some differences certainly exist between caterpillar gut microbial communities (because caterpillars were surface sterilized, we expect them to be enriched for gut microbes) and the microbial communities we sampled from frass, and these differences could affect some of our conclusions. Second, we failed to amplify DNA from about half of our samples (we purified DNA from 145 samples but only successfully sequenced microbes in 69 of them). We think that this reflects variation in the abundance of microbes, particularly in the low biomass frass samples from early instar caterpillars (where we had the least success). An effect of raw microbe abundance on amplification success could introduce some bias, but we would not expect it to bias results in terms of comparisons among treatments (i.e., raw microbe abundance does not appear to vary by treatment). Finally, it is almost certain that our sequence data include contaminant microorganisms, as (i) it is unlikely that the sterilization procedures fully eliminated non-target microbes, and (ii) microbes are also often present in DNA extraction kits and reagents^44^ (which is something that we cannot currently quantify based on our existing data). Nonetheless, our main interest was in differences among samples, and thus, we do not think that contaminants have created false positive signals (but they could have obscured true signals). In other words, contamination should alter microbial communities, but not create differences across treatments, particularly as all samples were processed with the same kit and reagents, in a haphazard order (with respect to all treatments), and in the same lab. With that said, lower biomass samples (e.g., frass from early instar larvae) could be more easily overwhelmed by contaminants. However, three lines of evidence suggest that this was not a major issue in this study: (i) lower biomass frass samples mostly failed to amplify (and thus did not contribute to our results), (ii) the major shift in microbial communities occurred between 20 and 25 days of development when larger frass and caterpillar samples were available, and (iii) we found many of the same common OTUs as have been found in other recent studies of Lepidopteran gut microbes (i.e., Proteobacteria and Firmicutes)^45^.

We had clear evidence that microbial community composition in *L*. *melissa* caterpillar guts shifted over time and exhibited a decrease in diversity. A similar pattern was recently found in *L*. *melissa* fungal communities^33^. Temporal variation in microbial communities has also been documented in *Heliconius* butterflies, but at different developmental stages (larvae versus pupae versus adults) rather than within a single developmental stage. Despite this temporal variation, several phylotypes were common across many of the *L*. *melissa* frass and larvae samples, including Actinobacteria, Proteobacteria and Bacilli (Firmicutes). Proteobacteria have frequently been found in other insects, including other Lepidoptera, plataspid bugs, alydid bugs, reed beetles, bees and termites^25, 45–47^. Proteobacteria could play a role in nutrient provisioning and degradation of toxins^47^. Our results show that *L*. *melissa* harbor all three classes of Proteobacteria (Alpha, Beta and Gamma). Alphaproteobacteria was also identified as a core bacteria in mosquito species with different diets^42^ and can be horizontally and vertically transferred^48, 49^. Fimicutes (Bacilli, order Lactobacillales) were also common in *L*. *melissa* frass and larvae. Firmicutes have been reported in other Lycaenid butterflies^37^, other Lepidopteran larvae^25, 45, 46, 50^, fruit flies and ground beetles^51, 52^. Firmicutes have been identified to play a role in nutrient provisioning, food digestion and fermentation^47^. Actinobacteria have also been reported in Lepidopteran larvae^25, 46^ and have been reported to play a role in nutrient provisioning^47^.

Neither diet (host plant speices) nor butterfly population (BST vs. HWR) had a detectable effect on overall microbial community composition. Nonetheless, diet was associated with the relative abundance of a few common microbes (Lactobacillales, Rhodospirillale, and Pseudomonadales), and similarly, a few microbes were more abundant in specific populations (Enterobacteriales, Sphingomondadales, and Rhizobiales). Diet has been shown to affect gut microbial communities in insects, including other Lepidoptera^25, 37, 46, 50^, bees^53^, *Drosophila* ^54^ and mosquitoes^42^. An effect of diet on microbial community has also been shown in mammals, including humans^34^, suggesting that this is a general mechanism shared by distantly related taxa. Thus, the fact that our results show an effect of diet on at least some microbes is unsurprising, and the limited nature of this effect might reflect similarities in the microbiomes of the two plant species (we lacked sufficient sample sizes to formally test for differences between plant species, but the communities from the *M*. *sativa* and *L*. *argenteus* plants overlapped in ordination space). In contrast, a recent study of Lycaenid butterflies failed to show a consistent effect of diet (including herbivory versus carnivory) on caterpillar gut microbiomes^37^. But, this study considered a few individuals across many different species of butterflies, so the lack of consistency is not evidence for a lack of an effect of diet within butterfly species.

Our results suggest that genetic differences between the two butterfly populations have at most a limited effect on the gut environment as perceived by most of the detected gut microbes. Perhaps this is unsurprising, as these populations occur in similar environments (mid-elevation, dry montane environments), have one of the same host plants (*M*. *sativa*; *L*. *argenteus* is used as an additional host at HWR) and are only separated by about 20 km. Thus, insufficient time, on-going gene flow or limited divergent selection could explain this lack of genetic divergence in microbial communities, and thus genetic divergence in microbial communities is still possible for more distant populations or those that differ more in host use. Likewise, genetic variation for gut microbiomes could exist within populations, but testing for this would require larger sample sizes (more families and caterpillars per family).

We failed to find compelling evidence for an association between larval performance (weight) and microbial community composition, though it is still possible that microbes are associated with other fitness components or metrics. Our results differ from a recent study that detected an association between growth and gut microbiome in the Glanville fritillary (*Melitaea cinxia*)^25^. Likewise, an association between microbial community and fitness has been detected in *Drosophila* and pea aphids^22, 23, 55^. But, in most of these cases (as would have been the case in our study) it is unclear whether the microbiome affects insect performance or insect performance affects the microbiome (i.e., different microbes could be favored in healthier insects) or both. Experiments whereby microbes are directly manipulated have been conducted in pea aphids to test these alternatives, but the results have been inconclusive^22–24^. Studies in humans suggest complex interactions between gut microbes and health that include feed-backs^56–59^. Similar complexity could exist in herbivorous insects.

In conclusion, we found that caterpillar gut microbial communities mostly varied with age and we failed to find convincing evidence that gut microbes play a role in host-plant adaptation in *L*. *melissa*. Other recent observational and experimental work on Lepidopterans suggests that this might be a general pattern^45^. Specifically, caterpillar gut microbiomes may be mostly transient, of low abundance and diversity, and have little to no affect on caterpillar performance. If so, Lepidopteran gut microbiomes would provide an interesting contrast to other systems where gut microbes are more critical.

## Methods

### Larval rearing experiment

In June of 2014, female *L*. *melissa* butterflies were captured at BST (N = 31) and HWR (N = 23) and caged individually in oviposition cages to lay eggs (as in ref. 60). After 48 hours, eggs were collected and stored in petri dishes at room temperature under bright lights until hatching. We obtained 182 neonate larvae from 20 of the females that layed eggs (i.e., not all females layed eggs; mean number of caterpillars per family = 9.1, s.d. = 9.6). Neonate larvae were transferred individually to new petri dishes once they hatched. Each caterpillar was fed exclusively on *M*. *sativa* (alfalfa) from BST or HWR, or *L*. *argenteus* from HWR. These diet treatments were assigned in alternation when caterpillars hatched. Fresh plant material was collected from the field once a week and fed to larvae *ad libitum* as small sprigs without flowers and with leaf petioles wrapped in damp Kimwipes. Petri dishes were checked and cleaned daily. Caterpillars were reared at room temperature on lab bench tops under 12-h light:dark cycles as we have done for other experiments with *Lycaeides* butterflies^29, 31^.

Petri dishes were checked daily to determine whether caterpillars were alive or dead, and survival time in days was recorded for dead caterpillars. As a second measure of performance, larval weight was quantified at 15, 20 and 25 days (weight was only measured for living caterpillars that had not yet pupated). Caterpillars were weighed on a Mettler Toledo XS64 microbalance to the nearest 0.1 mg (an average of three measurements was recorded). Frass was collected from each petri dish at 15, 20 and 25 days as well, and then stored by freezing at −80 °C in 1.5 mL tubes for subsequent microbial DNA extraction. A previous study with *Heliconius* butterflies showed that frass communities are a good proxy for gut microbial communities sampled from whole caterpillars^43^. Thus, frass samples can provide a non-lethal way to sample caterpillar gut microbes over time and without contamination from cellular endosymbionts commonly found in Lepidoptera, such as *Wolbachia* ^36, 37^. Nonetheless, more substantial differences between frass and gut communities could occur in some systems. Thus, 38 randomly chosen caterpillars were sacrificed and frozen at 15 (N = 14), 20 (N = 16) or 25 (N = 8) days so that gut microbial communities from caterpillars could be compared with the frass communities.

### DNA extraction and sequencing

DNA was isolated from the frass (N = 93) and whole caterpillar (N = 40) samples described above and from field-collected plant samples (N = 12, details follow). Prior to extraction, frozen caterpillars were surface-sterilized by rinsing them three times for one minute each in a 1% Sodium Hypochlorite (diluted bleach), 95% ethanol and deionized water (to rinse the samples). This was done to reduce the prevalence of surface microbes while leaving gut microbes intact. This procedure is unlikely to have removed all surface microbes, but it should enrich our samples for gut rather than surface microbes. Fresh leaf tissue was collected from *M*. *sativa* and *L*. *argenteus* at BST and HWR (leaves from four plants per site and species). Leaves were collected during the rearing experiment, so that they would be representative of the age and phenology of leaves being fed to the caterpillars. Surface microbes (epiphytes) were isolated by washing plant leaves in an isotonic 0.1 M PBS buffer. Bacterial cells were then extracted from the PBS buffer by passing the buffer through a Nalgene 0.2 micron vacuum filter; the filter paper was then cut into small pieces and used as a template for DNA extraction. We then sterilized the remaining leaf tissues by washing with 1% Sodium Hypochlorite, 95% ethanol and deionized water (as described above) and retained this tissue for isolation of endophytic microbial DNA.

Genomic DNA was extracted from frass, caterpillar and plant (epiphytes and endophytes) samples using the MoBio PowerSoil kit according to the manufacturers standard protocol. We then amplified the V4 region of the 16S rRNA gene using the standard PCR primers 515F and 806R and in accordance with the recommended protocol from the Earth Microbiome Project and as described previously in ref. 61. The primer design used included unique barcode sequences for index reads so that samples could be multiplexed. We successfully amplified DNA from 69 of the 145 samples. 16S rRNA amplicon libraries for these samples were sequenced at the University of Texas Genomic Sequencing and Analysis Facility (Austin, TX) on the Illumina MiSeq platform. We obtained 13.4 million 250-bp, paired-end reads.

### Identification of OTUs

We used the Quantitative Insight into Microbial Ecology (QIIME) pipeline to assign phylotypes (operational taxonomic units or OTUs) to 16S rRNA sequences and to estimate the relative abundance of each OTU in each sample^62^. We specifically used QIIME’s open reference-based OTU picking strategy^62^, which uses UCLUST to cluster sequences^63^. Sequences were first clustered against the Green Genes database (ver. 13-08) with a minimum of 97% sequence similarity^64^. A subset of sequences that did not cluster in this first step were clustered *de novo*, and the centroids of the new clusters were used to generate an additional sequence set for reference-based clustering (also at 97% sequence similarity). A final round of *de novo* clustering was conducted with sequences that did not match this reference sequence set. Taxonomic identifications were then assigned from the Green Genes database based on the centroid of each cluster^65^.

The 16S rRNA primers 515F and 806R also amplify chloroplast and mitochondrial DNA^66, 67^. Thus, sequences identified by the Green Genes database as chloroplast or mitochondrial 16S rRNA were removed before downstream analysis. We used rarefaction to ensure comparisons among microbial communities were not biased based on differences in the number of sequences obtained. Specifically, we randomly retained 1311 16S rRNA sequences from each sample for downstream analysis (this number was chosen as a compromise between removing samples and sequences). The samples which had fewer than 1311 bacterial sequences were removed from the analysis, which decreased our sample size from 69 samples to 59 samples: 41 frass communities, 10 caterpillar communities, and eight plant communities (five epiphyte and four endophyte samples). We also dropped unassigned OTUs before proceeding with our downstream analysis.

### Statistical analyses of community structure

All statistical analyses were conducted using the R statistical computing environment (R version 3.3.1^68^). We used two complementary ordination methods to summarize patterns of microbial community composition across the frass, caterpillar and plant (epiphytes and endophytes) samples (N = 59 communities). First, we conducted a principal component analysis (PCA) on the centered (but not scaled) chord-transformed relative abundance matrix. Chord transformation prior to PCA reduces the tendency for patterns to be driven by shared absences of microbes, while still allowing for ordination of (transformed) relative abundances^69, 70^. Second, we used principal coordinate analysis (PCOA) to ordinate samples based on pair-wise Bray-Curtis dissimilarities. The two methods gave very similar results (e.g., the Pearson correlation between PC1 and PCO1 scores was 0.98), and thus we mostly focus on the PC scores in the main text. Results based on PCOA are provided in the supplemental material. The ${\mathtt{prcomp}}$ function in ${\mathtt{R}}$ was used for PCA, the ${\mathtt{vegdist}}$ function in ${\mathtt{Vegan}}$ package was used to calculate the Bray-Curtis dissimilarities^71^, and the ${\mathtt{pcoa}}$ function in ape was used for PCOA^72^.

We next quantified the OTU diversity of microbial communities using Hill numbers^73, 74^. Specifically, we calculated the “true” diversity or effective number of phylotypes in each sample as ${}^{q}D={({\sum }_{i}{p}_{i}^{q})}^{1/(1-q)}$; *p*_*i*_ is the relative abundance of OTU *i* in the sample, and *q* determines the weight given to rare phylotypes. We chose *q* = 2, as lower values are unlikely to yield reliable estimates of diversity for microbial communities^75^. In addition we calculated effective number of species per sample using the same diversity index to determine the magnitude of diversity retained for each sample as sequencing depth increases.

We used Bayesian models to quantify differences in microbial community composition (PC1, PC2, PCO1 and PCO2 scores) and diversity (^*q*=2^ *D*) among sample types, that is, frass, caterpillar and plant communities (ephiphytes and endophytes were pooled because of small sample sizes and because visual inspection of PCA plots suggested these communities were similar). The model we used can be viewed as a Bayesian analog to a single factor ANOVA, but without the constraint of equal variances across treatments. For each metric (PC1, PC2, PCO1, PCO2 and ^2^ *D*) we assumed that the scores or values from each sample type could be characterized by a Normal distribution with an unknown mean and standard deviation (s.d.) that we estimated from the data. We placed uninformative priors on the mean (Normal, with *μ* = 0, and $\tau =\tfrac{1}{{\sigma }^{2}}=1e-6$) and precision (that is, the inverse of the variance; gamma with shape and rate parameters equal to 0.01) for each group.

We then used a classification method, Random Forest (RF), to determine whether frass, whole caterpillar and plant communities could be assigned to their respective sample type and if so, to identify the microbes or combination of microbes that were most important for accurate classification. RF was performed in R with the ${\mathtt{randomForest}}$ function using 50,000 trees^76^. An advantage of this approach (e.g., compared to discriminant analysis) is that the number of observations (samples) does not have to be larger than the number of variables used for classification (i.e., the number of microbial OTUs).

We found that the microbial communities from frass and whole caterpillar samples differed, mostly because the intracellular endoysmbiont *Wolbachia* (Order Rickettsiales) was highly abundant in the caterpillars but almost completely absent from the frass (see Results for details). *Wolbachia* is not a free-living member of the gut microbial community, but rather a vertically transmitted intracellular endosymbiont found in germ and somatic tissues^77^. Thus, we removed the *Wolbachia* sequences before continuing with tests of the determinants of gut microbial community. Specifically, we re-rarefied the original OTU table after (i) dropping chloroplast, mitochondrial and *Wolbachia* sequences, (ii) removing the plant samples (which were not needed for additional analyses), and (iii) dropping rare OTUs with a relative abundance of <1% in all samples. For this rarefaction, we randomly retained 500 sequences from each sample for downstream analysis. This decreased our sample size to 53 samples (42 frass communities and 11 caterpillar communities). Note that one frass and one caterpillar retained in this round of rarefaction were dropped from the first set of analysis as the minimum number of sequences required for retention was different. We again removed any unassigned OTUs before proceeding with downstream analysis.

### Determinants of frass and caterpillar microbial communities

We investigated the effect of diet, population and caterpillar age on the microbial communities detected in the frass and whole caterpillar samples (N = 53) using the re-rarefied OTU data. Using both frass and whole caterpillar samples allowed us to increase our sample size and assess remaining differences between these sample types after removing *Wolbachia* sequences. We first used a hierarchical clustering approach to test the relatedness of bacterial communities in frass and larvae. We used Bray-Curtis dissimilarities (recommended for relative abundance data) and the Unweighted Pair Group Method with Arithmetic Mean (UPGMA) method for clustering to determine relatedness in bacterial communities in frass and larvae samples^78, 79^. The vegdist function in Vegan package in R was used to calculate the dissimilarity matrix^71^. Hierarchical Clustering was performed by using the hclust function in R^80^. heatmap.2 function from gplots package in ${\mathtt{R}}$ was used to visualize the distance matrix and clustering results^81^.

We then calculated phylotype diversity (^2^ *D*) for each frass and whole caterpillar sample and conducted a second PCA and PCOA to summarize microbial community patterns across these samples. Bayesian linear models were used to quantify the effect of sample type (frass or whole caterpillar), plant, population (all binary covariates), and days since hatching (i.e., larval age or development) on microbial community composition (PC1, PC2, PCO1 and PCO2 scores) and diversity (^2^ *D*). Uninformative priors were placed on the regression coefficients, *β*_*type*_, *β*_*plant*_, *β*_*pop*_ and *β*_*age*_ (Normal, with *μ* = 0, and $\tau =\tfrac{1}{{\sigma }^{2}}=1e-6$) and on the precision term (gamma with shape = 0.01 and rate = 0.01).

We also tested for differences in the relative abundance of individual microbes. We did this by fitting Bayesian multinomial-Dirichlet models for the rarefied OTU count data. This model assumes that microbe counts for each sample are drawn from a multinomial distribution with success probabilities (*π*) give by the true relative abundances. We placed an uninformative prior on the vector of true relative abundances (*π*~Dirichlet(1, …, 1)). As this model does not readily incorporate covariates, we focused on a more homogeneous subset of the data: frass samples at 15 to 20 days. This minimized the effects of age and sample type while maximizing the sample size: *n* = 35, which includes frass from 12 HWR caterpillars reared on *L*. *argenteus*, 10 HWR caterpillars reared on *M*. *sativa* and 13 BST caterpillars reared on *M*. *sativa* (we removed the single sample from a BST caterpillar reared on *L*. *argenteus* that otherwise met the criteria for inclusion as the sample size, *n* = 1, was too low for valid inference). We fit and compared models (i) allowing the true relative abundance vector (*π*) to vary among the three source population × host plant (diet) treatments, and (ii) constraining the true relative abundances to be the same (i.e., a null model assuming no differences in microbiome among samples). We used the posterior predictive distribution of the count data (predicted OTU counts from the model) to measure model performance by calculating the root-mean square error between the observed and predicted counts.

Bayesian parameter estimates were obtained using Markov Chain Monte Carlo (MCMC) via the R interface with JAGS provided by the rjags package^82^ (most analyses) or by direct simulation from the closed form posterior in R (the multinomial-Dirichlet model has a closed form solution and can be directly sampled). Three replicate MCMC runs (chains) were used for each analysis. Each chain included a 1000 iteration burn-in followed by 10,000 iterations where every third sample was retained for inference. Adequate MCMC mixing and likely convergence to the stationary distribution were verified by quantifying the effective sample size and calculating the Gelman Rubin convergence diagnostic. We used the median of the marginal posterior distribution for each parameter as a point estimate (denoted ‘pm’ for posterior median). Uncertainty in parameter estimates was quantified based on marginal 95% equal-tail probability intervals [ETPIs], and, in some cases, based on the posterior probability (hereafter, ‘pp’) that a parameter or the difference between two parameters was greater than or less than 0. Finally, we re-ran the RF classification analysis to determine whether frass and whole caterpillar communities could be assigned to sample type (frass vs. whole caterpillar), diet/host plant (*M*. *sativa* vs. *L*. *argenteus*), population (BST vs. HWR) and caterpillar age (15 vs. 20 vs. 25 days), and if so, to identify the microbes or combination of microbes that were most important for accurate classification.

### Tests for an effect of gut microbiome on larval performance

We fit Bayesian models to quantify the effect of food plant (diet) and butterfly population on larval performance and to determine whether microbial community explained additional variation in performance. First, we used a Bayesian generalized linear model with a Bernouli error distribution and logit link function to quantify the effect of plant (*β*_plant_) and population (*β*_pop_) on caterpillar survival to 15 days (that is the time when the first frass sample was taken). Survival data from 181 caterpillars were used for this analysis. Next, we fit and compared alternative models for caterpillar weight. All models included a potential effect of plant (*β*_plant_), population (*β*_pop_) and time since hatching (larval age; only 15 and 20 day-old caterpillars were included, as the sample size for 25 day caterpillars was very small and these caterpillars were much larger; *β*_age_). We considered models with just these effects or these effects plus measures of microbial community composition from frass samples collected at 15 or 20 days (PC1, PC2, PCO1 and PCO2 scores) or community diversity (^2^ *D*). We tested for possible interactions between microbial community and diet (plant) on weight, which would suggest a potential role of microbes in host-specific adaptation. For all models, uninformative priors were placed on the regression coefficients, *β*_*type*_, *β*_*plant*_, *β*_*pop*_, *β*_*age*_ and *β*_*PC*_, *β*_*PCO*_ or ${\beta }_{{}^{2}D}$ (Normal, with *μ* = 0, and $\tau =\tfrac{1}{{\sigma }^{2}}=1e-6$) and on the precision term (gamma with shape = 0.01 and rate = 0.01). Model comparisons were based on deviance information criterion (DIC), which is similar to Akaikie information criterion but appropriately penalizes Bayesian models based on the effective number of parameters (this is readily calculated from MCMC output).

## Electronic supplementary material

Supplemental material
