# Supplementary material for: Sources of Variation in the Gut Microbial Community of Lycaeides melissa Caterpillars
Source: Sci Rep. 2017 Sep 12;7:11335. doi: 10.1038/s41598-017-11781-1 (PMC5595848; doi:10.1038/s41598-017-11781-1)
Supplement: Supplementary file 1 — Supplemental material [file 41598_2017_11781_MOESM1_ESM.pdf]

# Sources of Variation in the Gut Microbial Community of *Lycaeides melissa* Caterpillars

**Samridhi Chaturvedi[1,2], Alexandre Rego[1], Lauren K. Lucas[1], and Zachariah Gompert[1,2,*]**

[1]Utah State University, Department of Biology, Logan, 84322, USA
[2]Utah State University, Ecology Center, Logan, 84322, USA
[*]zach.gompert@usu.edu

# Supplemental Material

## Supplemental Tables and Figures

**Table S1.** PC1 and PC2 loadings for top microbial phylotypes in epiphytes, endophytes, frass and larvae after removing chloroplast and mitochondria and following chord transformation of the relative abundance data.

| Microbe class | PC1 rotations for epiphytes, endophytes, frass and larvae |
|---|---|
| Gammaproteobacteria (Xanthomonadales) | 0.49 |
| Betaproteobacteria (Burkholderiales) | 0.49 |
| Alphaproteobacteria (Sphingomonadales) | 0.47 |
| Alphaproteobacteria (Caulobacterales) | 0.12 |
| Bacilli (Bacillales) | 0.02 |
| **Microbe class** | **PC2 rotations for epiphytes, endophytes, frass and larvae** |
| Gammaproteobacteria (Pseudomonadales) | 0.89 |
| Bacilli (Bacillales) | 0.04 |
| Alphaproteobacteria (Rhodobacterales) | 0.01 |
| Gammaproteobacteria (Xanthomonadales) | 0.006 |
| Sphingobacteriia (Sphingobacteriales) | 0.005 |

**Table S2.** Random Forest confusion matrix for correct assignment of plant, frass and larvae microbial communities to sample type (Out of Bag (OOB) estimate of error rate = 16.67%). Rows indicate actual class and columns indicate predicted class.

| Type | Frass | Larvae | Plant | error |
|---|---|---|---|---|
| Frass | 41 | 0 | 0 | 0.0 |
| Larvae | 2 | 8 | 0 | 0.20 |
| Plant | 8 | 0 | 1 | 0.88 |

**Table S3.** Top five microbial phylotypes in frass, larvae and plants based on importance assigned by Random Forest (RF) GINI Indexes for class sample type.

| Microbe class | RF GINI Index - Sample type |
|---|---|
| Alphaproteobacteria (Rickettsiales) | 4.89 |
| Betaproteobacteria (Burkholderiales) | 2.46 |
| Gammaproteobacteria (Xanthomonadales) | 1.85 |
| Alphaproteobacteria (Caulobacterales) | 1.83 |
| Alphaproteobacteria (Sphingomonadales) | 1.17 |
| Bacilli (Bacillales) | 1.08 |

**Table S4.** PC1 and PC2 loadings for top microbial phylotypes in frass and larvae after removing *Wolbachia* and following chord transformation of the relative abundance data.

| Microbe class | PC1 rotations for frass and larvae |
|---|---|
| Betaproteobacteria (Burkholderiales) | 0.48 |
| Alphaproteobacteria (Sphingomonadales) | 0.46 |
| Gammaproteobacteria (Xanthomonadales) | 0.45 |
| Alphaproteobacteria (Caulobacterales) | 0.12 |
| Actinobacteria (Actinomycetales) | 0.05 |
| **Microbe class** | **PC2 rotations for frass and larvae** |
| Gammaproteobacteria (Enterobacteriales) | 0.56 |
| Alphaproteobacteria (Sphingomonadales) | 0.15 |
| Betaproteobacteria (Burkholderiales) | 0.05 |
| Actinobacteria (Actinomycetales) | 0.04 |
| Alphaproteobacteria (Caulobacterales) | 0.03 |

**Table S5.** Random Forest Out of Bag (OOB) estimate of error rate for classes after removing *Wolbachia*.

| Class | OOB error rate |
|---|---|
| Type | 16.98% |
| Age | 32.08% |
| Age (15 + 20 days) | 26.42% |
| Plant | 39.62% |
| Population | 45.28% |

**Table S6.** Confusion matrixes for Random Forest assignment of samples based on microbial community for frass and larvae after removing *Wolbachia*. Results are shown for age (with or without combining 15 and 20 days), sample type (frass or whole caterpillar), host plant species (alflafa or lupine) and populations (BST or HWR).

| Age | 15days | 20 days | 25 days | Error |
|---|---|---|---|---|
| 15 | 29 | 0 | 2 | 0.06 |
| 20 | 9 | 0 | 2 | 1 |
| 25 | 4 | 0 | 7 | 0.36 |
| **Age** | **15 + 20 days** | **25 days** | **Error** | |
| 15 + 20 | 38 | 4 | 0.09 | |
| 25 | 10 | 1 | 0.91 | |
| **Type** | **Frass** | **Larvae** | **Error** | |
| Frass | 41 | 1 | 0.02 | |
| Larvae | 8 | 3 | 0.72 | |
| **Plant** | **Lupine** | **Alfalfa** | **Error** | |
| Lupine | 3 | 15 | 0.83 | |
| Alfalfa | 6 | 29 | 0.17 | |
| **Population** | **BST** | **HWR** | **Error** | |
| BST | 8 | 15 | 0.65 | |
| HWR | 9 | 21 | 0.30 | |

**Table S7.** Top five microbial phylotypes in frass and larvae based on importance assigned by Random Forest (RF) GINI Indexes for classes sample type, age and plant.

| Microbe class | RF GINI Index - Sample type |
|---|---|
| Betaproteobacteria (Burkholderiales) | 1.96 |
| Gammaproteobacteria (Xanthomonadales) | 1.68 |
| Alphaproteobacteria (Caulobacterales) | 1.65 |
| Alphaproteobacteria (Sphingomonadales) | 1.11 |
| Bacilli (Lactobacillales) | 1.07 |
| **Microbe class** | **RF GINI Index - Age (15 + 20 vs 25)** |
| Gammaproteobacteria (Enterobacteriales) | 2.39 |
| Betaproteobacteria (Burkholderiales) | 1.88 |
| Gammaproteobacteria (Pseudomonadales) | 1.49 |
| Alphaproteobacteria (Sphingomonadales) | 1.19 |
| Alphaproteobacteria (Caulobacterales) | 1.12 |
| **Microbe class** | **RF GINI Index - Age (15 vs 20 vs 25)** |
| Betaproteobacteria (Burkholderiales) | 2.97 |
| Gammaproteobacteria (Enterobacteriales) | 2.77 |
| Alphaproteobacteria (Sphingomonadales) | 2.03 |
| Gammaproteobacteria (Pseudomonadales) | 1.93 |
| Bacilli (Bacillales) | 1.90 |
| **Microbe class** | **RF GINI Index - Plant** |
| Gammaproteobacteria (Oceanospirillales) | 2.51 |
| Gammaproteobacteria (Xanthomonadales) | 1.81 |
| Gammaproteobacteria (Pseudomonadales) | 1.75 |
| Alphaproteobacteria (Sphingomonadales) | 1.69 |
| Betaproteobacteria (Burkholderiales) | 1.54 |
| **Microbe class** | **RF GINI Index - Population** |
| Gammaproteobacteria (Enterobacteriales) | 2.49 |
| Gammaproteobacteria (Xanthomonadales) | 1.80 |
| Gammaproteobacteria (Pseudomonadales) | 1.58 |
| Actinobacteria (Actinomycetales) | 1.54 |
| Alphaproteobacteria (Sphingomonadales) | 1.44 |

**Table S8.** Model comparison for the association of microbial community with larval weight ($\bar{D}$ = mean deviance, pD = effective number of parameters, $\Delta$ DIC = difference in DIC compared to the best model).

| Model | $\bar{D}$ | pD | $\Delta$ DIC |
|---|---|---|---|
| null model | 69.0 | 5.4 | 0 |
| PC1 | 70.2 | 6.5 | 2.2 |
| PC1 $\times$ plant | 68.7 | 7.6 | 2.0 |
| PC2 | 70.2 | 6.5 | 2.4 |
| PC2 $\times$ plant | 70.7 | 7.6 | 4.0 |
| PCO1 | 69.8 | 6.5 | 2.0 |
| PCO1 $\times$ plant | 68.4 | 7.6 | 1.8 |
| PCO2 | 70.2 | 6.5 | 2.3 |
| PCO2 $\times$ plant | 70.2 | 7.6 | 3.5 |
| $^2D$ | 70.2 | 6.5 | 2.4 |
| $^2D$ $\times$ plant | 71.4 | 7.6 | 4.1 |

**Table S9.** Microbial phylotypes found across frass, larvae, endophyte and epiphyte samples after removing chloroplast and mitochondria. In some cases microbes lack formal taxonomic IDs at lower levels (e.g., Class and Order).

| OTU Number | Mean relative abundance | Phylum | Class | Order |
|---|---|---|---|---|
| 1 | 7.00E-5 | Crenarchaeota | Thaumarchaeota | Nitrososphaerales |
| 2 | 0.00003 | Euryarchaeota | Halobacteria | Halobacteriales |
| 3 | 0.0004 | Acidobacteria | Acidobacteria | Iii1.15 |
| 4 | 4.00E-5 | Acidobacteria | Acidobacteriia | Acidobacteriales |
| 5 | 0.00002 | Acidobacteria | S035 | - |
| 6 | 0.0002 | Acidobacteria | Chloracidobacteria | RB41 |
| 7 | 1.00E-5 | Acidobacteria | iii1.8 | DS.18 |
| 8 | 0.0002 | Actinobacteria | Acidimicrobiia | Acidimicrobiales |
| 9 | 0.03 | Actinobacteria | Actinobacteria | Actinomycetales |
| 10 | 0.0005 | Actinobacteria | Actinobacteria | Bifidobacteriales |
| 11 | 0.0003 | Actinobacteria | Coriobacteriia | Coriobacteriales |
| 12 | 1.00E-5 | Actinobacteria | Nitriliruptoria | Nitriliruptorales |
| 13 | 6.00E-5 | Actinobacteria | Rubrobacteria | Rubrobacterales |
| 14 | 4.00E-5 | Actinobacteria | Thermoleophilia | Gaiellales |
| 15 | 0.0002 | Actinobacteria | Thermoleophilia | Solirubrobacterales |
| 16 | 2.00E-5 | Bacteroidetes | - | - |
| 17 | 3.00E-5 | Bacteroidetes | BME43 | - |
| 18 | 0.001 | Bacteroidetes | Bacteroidia | Bacteroidales |
| 19 | 0.001 | Bacteroidetes | Cytophagia | Cytophagales |
| 20 | 0.002 | Bacteroidetes | Flavobacteriia | Flavobacteriales |
| 21 | 0.004 | Bacteroidetes | Sphingobacteriia | Sphingobacteriales |
| 22 | 2.00E-5 | Bacteroidetes | Rhodothermi | Rhodothermales |
| 23 | 0.001 | Bacteroidetes | Saprospirae | Saprospirales |
| 24 | 9.00E-6 | Chlamydiae | Chlamydiia | Chlamydiales |
| 25 | 0.0003 | Chloroflexi | Anaerolineae | SBR1031 |
| 26 | 1.00E-5 | Chloroflexi | Chloroflexi | AKIW781 |
| 27 | 4.00E-5 | Chloroflexi | Ellin6529 | - |
| 28 | 7.00E-5 | Chloroflexi | Gitt.GS.136 | - |
| 29 | 4.71E-5 | Chloroflexi | TK10 | AKYG885 |
| 30 | 0.0001 | Chloroflexi | Thermomicrobia | - |
| 31 | 0.0001 | Chloroflexi | Thermomicrobia | JG30.KF.CM45 |
| 32 | 0.00008 | Cyanobacteria | 4C0d.2.o | MLE1.12 |
| 33 | 1.00E-5 | Cyanobacteria | 4C0d.2 | YS2 |
| 34 | 4.00E-5 | Cyanobacteria | Oscillatoriophycideae | Oscillatoriales |
| 35 | 0.0001 | FBP | - | - |
| 36 | 1.00E-6 | Firmicutes | Bacilli | - |
| 37 | 0.02 | Firmicutes | Bacilli | Bacillales |
| 38 | 0.0002 | Firmicutes | Bacilli | Gemellales |
| 39 | 0.02 | Firmicutes | Bacilli | Lactobacillales |
| 40 | 0.006 | Firmicutes | Clostridia | Clostridiales |
| 41 | 0.0002 | Firmicutes | Clostridia | Thermoanaerobacterales |
| 42 | 0.002 | Firmicutes | Erysipelotrichi | Erysipelotrichales |
| 43 | 0.0003 | Fusobacteria | Fusobacteriia | Fusobacteriales |
| 44 | 2.00E-5 | Gemmatimonadetes | Gemm.1 | - |
| 45 | 0.0002 | Gemmatimonadetes | Gemm.3 | - |
| 46 | 1.00E-6 | Gemmatimonadetes | Gemm.5 | - |
| 47 | 1.00E-5 | Gemmatimonadetes | Gemmatimonadetes | - |
| 48 | 0.0003 | Gemmatimonadetes | Gemmatimonadetes | Gemmatimonadales |
| 49 | 3.00E-5 | Nitrospirae | Nitrospira | Nitrospirales |
| 50 | 0.00006 | OD1 | ZB2 | - |

Continued on next page

**Table S9 – continued from previous page**

| OTU Number | Mean relative abundance | Phylum | Class | Order |
|---|---|---|---|---|
| 51 | 0.0001 | Planctomycetes | Phycisphaerae | WD2101 |
| 52 | 5.00E-5 | Planctomycetes | Planctomycetia | Gemmatales |
| 53 | 2.00E-5 | Planctomycetes | Planctomycetia | Pirellulales |
| 54 | 7.00E-5 | Planctomycetes | Planctomycetia | Planctomycetales |
| 55 | 0.02 | Proteobacteria | Alphaproteobacteria | Caulobacterales |
| 56 | 0.01 | Proteobacteria | Alphaproteobacteria | Rhizobiales |
| 57 | 0.003 | Proteobacteria | Alphaproteobacteria | Rhodobacterales |
| 58 | 0.0008 | Proteobacteria | Alphaproteobacteria | Rhodospirillales |
| 59 | 0.05 | Proteobacteria | Alphaproteobacteria | Rickettsiales |
| 60 | 0.08 | Proteobacteria | Alphaproteobacteria | Sphingomonadales |
| 61 | 0.00003 | Proteobacteria | Betaproteobacteria | - |
| 62 | 0.1 | Proteobacteria | Betaproteobacteria | Burkholderiales |
| 63 | 0.0001690141 | Proteobacteria | Betaproteobacteria | Ellin6067 |
| 64 | 1.00E-5 | Proteobacteria | Betaproteobacteria | MND1 |
| 65 | 0.0001 | Proteobacteria | Betaproteobacteria | Methylophilales |
| 66 | 0.0005 | Proteobacteria | Betaproteobacteria | Neisseriales |
| 67 | 0.0003 | Proteobacteria | Betaproteobacteria | Rhodocyclales |
| 68 | 0.0001 | Proteobacteria | Betaproteobacteria | SC.I.84 |
| 69 | 8.00E-5 | Proteobacteria | Deltaproteobacteria | Bdellovibrionales |
| 70 | 0.0007 | Proteobacteria | Deltaproteobacteria | Myxococcales |
| 71 | 5.00E-5 | Proteobacteria | Epsilonproteobacteria | Campylobacterales |
| 72 | 0.0002 | Proteobacteria | Gammaproteobacteria | Aeromonadales |
| 73 | 0.0006 | Proteobacteria | Gammaproteobacteria | Alteromonadales |
| 74 | 0.1 | Proteobacteria | Gammaproteobacteria | Enterobacteriales |
| 75 | 0.004 | Proteobacteria | Gammaproteobacteria | Oceanospirillales |
| 76 | 0.007 | Proteobacteria | Gammaproteobacteria | Pasteurellales |
| 77 | 0.1 | Proteobacteria | Gammaproteobacteria | Pseudomonadales |
| 78 | 0.0003 | Proteobacteria | Gammaproteobacteria | Thiotrichales |
| 79 | 2.00E-5 | Proteobacteria | Gammaproteobacteria | Vibrionales |
| 80 | 0.1 | Proteobacteria | Gammaproteobacteria | Xanthomonadales |
| 81 | 1.00E-5 | Synergistetes | Synergistia | Synergistales |
| 82 | 0.0003 | TM7 | SC3 | - |
| 83 | 0.00007 | TM7 | TM7.3 | I025 |
| 84 | 0.0002 | Verrucomicrobia | Opitutae | Opitutales |
| 85 | 4.00E-5 | Verrucomicrobia | Verrucomicrobiae | Verrucomicrobiales |
| 86 | 0.00006 | Verrucomicrobia | Spartobacteria | Chthoniobacterales |
| 87 | 0.0001 | Thermi | Deinococci | Deinococcales |
| 88 | 5.00E-5 | Thermi | Deinococci | Thermales |

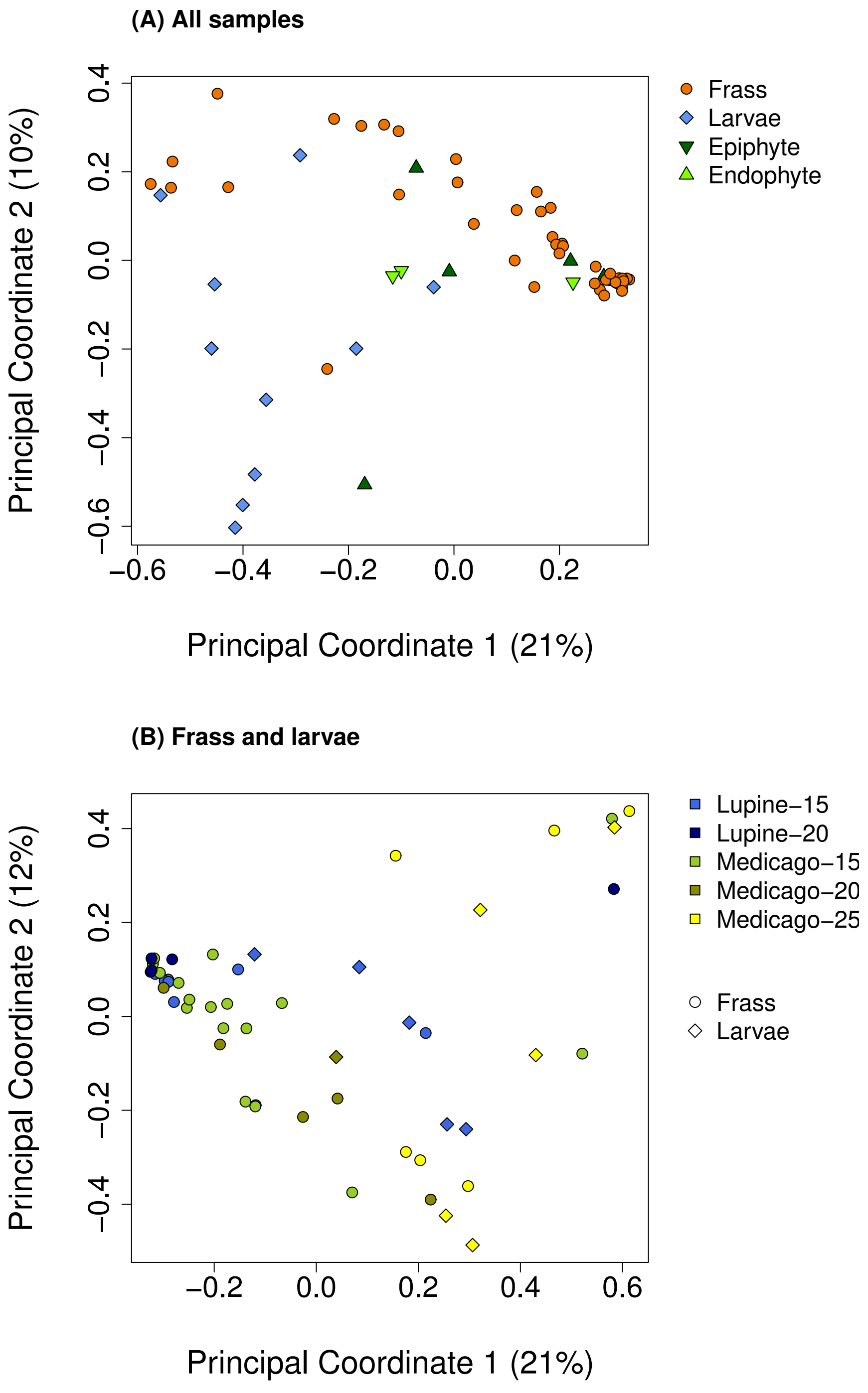


**Figure S1. Principal coordinate analysis.** Scatterplots show an ordination of microbial communities based on Bray-Curtis community dissimilarities for (A) all samples, or (B) frass and larvae. Colors and symbols denote different treatments and sample types (see legends). Caterpillar, frass and plant microbial communities overlapped in principal coordinate (PCO) space, but sample types differed in their average PCO scores and degree of variability (Table 1). Most notably, average caterpillar communities differed from frass and plant communities with respect to PCO1 scores (Bayesian posterior prob. [pp] $\mu_{\text{Larvae}} > \mu_{\text{Frass}} > 0.99$; pp $\mu_{\text{Larvae}} > \mu_{\text{Plant}} > 0.99$), and caterpillar and frass communities differed with respect to PCO2 scores (pp $\mu_{\text{Larvae}} < \mu_{\text{Frass}} = 0.99$). Frass and plant microbe communities were more similar.

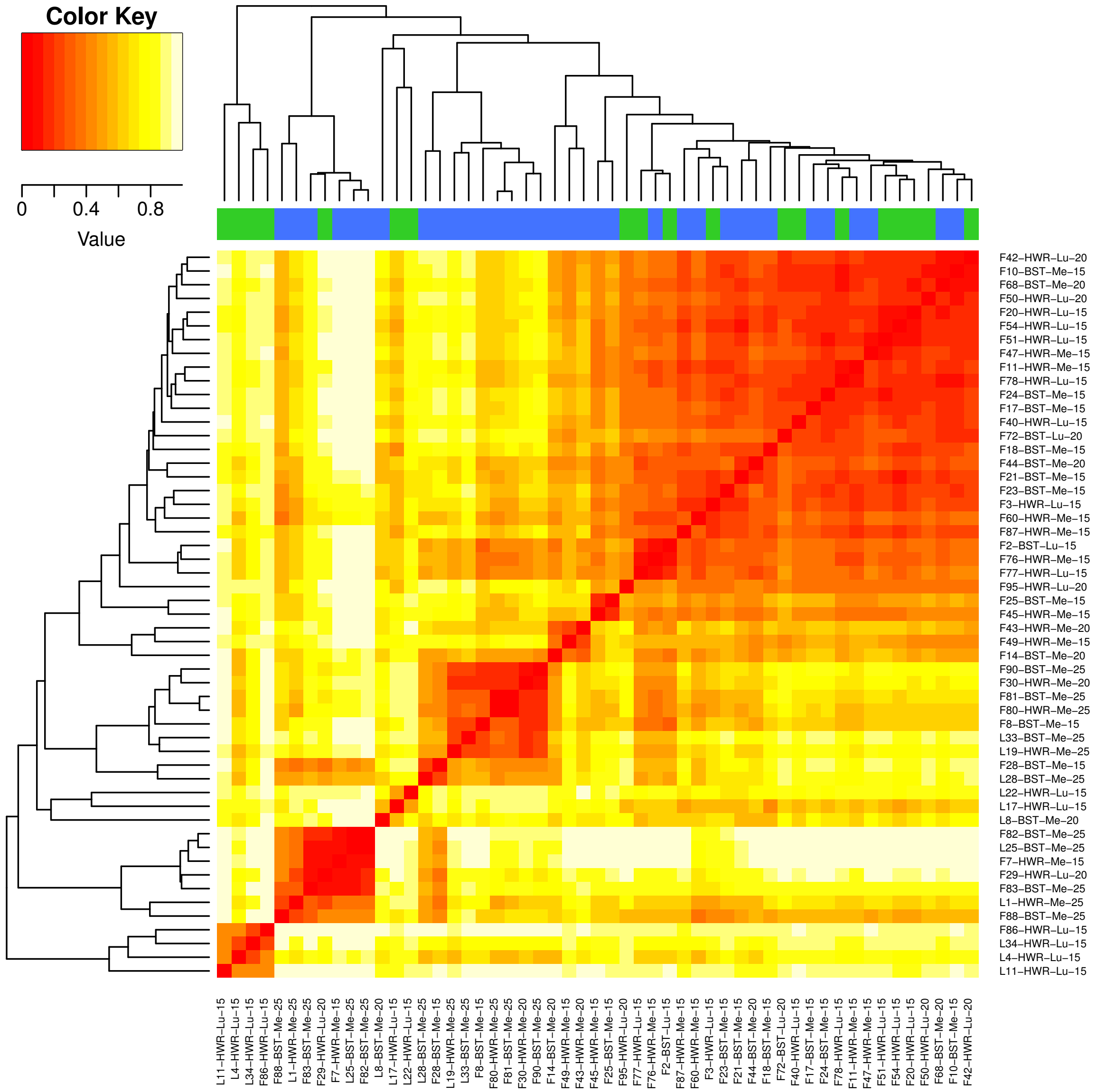


**Figure S2. Hierarchical Cluster Analysis.** Heatmap of Bray-Curtis dissimilarities between samples and corresponding dendrograms based on bacterial OTU abundances. Each row and column represents a sample. Cell colors indicate dissimilarity values between row and column microbial communities (red = greater similarity and yellow = less similarity). The dendrogram groups samples by heirarchical clustering based on microbial community similarity. Sample abbreviations: F = frass, L = larvae, HWR = Hardware Ranch, BST = Bonneville Shoreline Trail, Me = *M. sativa*, and Lu = *L. argenteus*. Numbers with F and L indicate sample IDs, whereas the final number in each ID gives the caterpillar age (15, 20, or 25). Color bars above the heatmap indicate the host plant each sample was reared on (green = *L. argenteus*, blue = *M. sativa*).. The heatmap and dendrogram show that microbiomes from different sample types, different age caterpillars and different treatments do not form distinct groups or clades, but that there is a tendency for sets of similar samples (i.e., samples from the same age caterpillar) to be more similar and cluster together. Note, this figure is identical to Fig. 4 except that colored bars denote plant rather than caterpillar age.
